# THE M-RNA, EXPRESSION OF SERCA2 AND NCX1 IN THE PROCESS OF PHARMACOLOGICAL CELL PROTECTION IN EXPERIMENTAL ACUTE PANCREATITIS INDUCED BY TAUROCHOLATE

**DOI:** 10.1590/0102-672020180001e1352

**Published:** 2018-06-21

**Authors:** Enio Rodrigues VASQUES, José Eduardo Monteiro CUNHA, Marcia Saldanha KUBRUSLY, Ana Maria COELHO, Sandra N. SANPIETRI, Helena B. NADER, Ivarne L.S. TERSARIOL, Marcelo A. LIMA, Eleazar CHAIB, Luiz Augusto Carneiro D’ALBUQUERQUE

**Affiliations:** 1 Gastroenterology Department LIM 37, University of São Paulo Medical School; 2 Pharmacology Department of Federal University of São Paulo, São Paulo, SP, Brazil

**Keywords:** Pancreatitis, Cytoprotection, Heparin, low-molecular-weight., Pancreatite, Citoproteção, Heparina de baixo peso molecular

## Abstract

**Background:**

Intracellular calcium overload is known to be a precipitating factor of pancreatic cell injury in acute pancreatitis (AP). Intracellular calcium homeostasis depends of Plasmatic Membrane Calcium ATPase (PMCA), Sarcoplasmic Endothelial Reticulum Calcium ATPase 2 (SERCA 2) and the Sodium Calcium Exchanger (NCX1). The antioxidant melatonin (Mel) and Trisulfate Disaccharide (TD) that accelerates NCX1 action could reduce the cell damage determined by the AP.

**Aim:**

To evaluate m-RNA expressions of SERCA2 and NCX1 in acute pancreatitis induced by sodium taurocholate in Wistar rats pre-treated with melatonin and/or TD.

**Methods:**

Wistar rats were divided in groups: 1) without AP; 2) AP without pre-treatment; 3) AP and Melatonin; 4) AP and TD; 5) AP and Melatonin associated to TD. Pancreatic tissue samples were collected for detection of SERCA2 and NCX1 m-R NA levels by polymerase chain reaction (PCR).

**Results:**

Increased m-RNA expression of SERCA2 in the melatonin treated group, without increase of m-RNA expression of the NCX1. The TD did not affect levels of SERCA2 and NCX1 m-RNA expressions. The combined melatonin and TD treatment reduced the m-RNA expression of SERCA2.

**Conclusions:**

The effect of melatonin is restricted to increased m-RNA expression of SERCA2. Although TD does not affect gene expression, its action in accelerating calcium exchanger function can explain the slightest expression of SERCA2 m-RNA when associated with Melatonin, perhaps by a joint action of drugs with different and but possibly complementary mechanisms.

## RESUMO

**Racional:**

A lesão celular da pancreatite aguda (PA) envolve sobrecarga de cálcio, regulada pela atividade da Cálcio ATPase de membrana (PMCA), Cálcio ATPase do Retículo (SERCA2) e pelo Trocador Sódio Cálcio (NCX1). A melatonina (antioxidante) e o Dissacarídeo Trissulfatado (acelerador do NCX1) poderiam reduzir a lesão celular na PA.

**Objetivo:**

Avaliar a expressão do RNAm da SERCA2 e NCX1 em modelo animal de pancreatite aguda tratados com melatonina e/ou dissacarídeo trissulfatado (DT).

**Método:**

Ratos Wistar foram divididos em grupos: 1) sem pancreatite aguda; 2) com pancreatite aguda por taurocolato; 3) PA e Melatonina; 4) PA e DT; 5) PA e Melatonina com DT. Amostras de tecido foram colhidas para detecção dos níveis de RNAm da SERCA2 e NCX1 por PCR.

**Resultados:**

Houve aumento da expressão do RNAm da SERCA2 no grupo com PA tratados com Melatonina, porém sem aumento de expressão do NCX1. O DT não afetou os níveis de SERCA2 e NCX1. O tratamento conjunto com Melatonina e DT diminuiu a expressão da SERCA2.

**Conclusões:**

O efeito da Melatonina é restrito ao aumento da expressão da SERCA2. O DT não tem ação na expressão gênica, porém sua ação na aceleração do trocador na retirada do cálcio pode explicar a menor expressão da SERCA2 quando associado à Melatonina, pela ação conjunta de drogas com mecanismos diferentes e possivelmente complementares.

## INTRODUCTION

Acute pancreatitis (AP) is an acute pancreatic inflammatory process resulting from inappropriately activation of pancreatic enzymes determining local and systemic effects. The acinar cells correspond to 90% of exocrine pancreatic tissue and are the first place to be committed^21^. Calcium has a significant role in the pathogenesis of AP induced by factors such as cerulein^18^, bile salts^20^ and^4^ fatty acids leading to early activation of intracellular zymogen granules, with vacuolation and cell death^18^. The stimulation of acinar cells by acetylcholine and cholecystokinin (CCK-8) elevates intracellular calcium concentration in a physiological form. In taurocholate-induced AP, there is an intracellular calcium release from endoplasmic reticulum via inositol 1, 4, 5, triphosphate (IP3) formation and ryanodine receptors (RyR), leading to merging of zymogen granules with the plasma membrane, determining the exocytosis of inactive precursor of digestive enzymes into the pancreatic tissue^10^. The exacerbation of this phenomenon with acinar cells hyper stimulation by CCK-8 increases the levels of calcium in the cytoplasm, intracellular premature activation of with digestive enzymes and cellular necrosis^18^. This phenomenon also occurs with bile salts and non-oxidative ethanol metabolites^5^ ^,^ ^6^. Since mitochondria also acts as a calcium buffer there is also a mitochondrial function impairment. The increased calcium levels leads to mitochondrial dysfunction, and decreased ATP production leading to damage of ATP-dependent calcium pumps, that contributes to the intracellular ion overload^17^ which, when sustained, leads to cell apoptosis. Intracellular ATP supplementation decreases this intracellular Ca elevation with consequent reduction of necrosis, as noted in pancreatitis caused by non-oxidative metabolites of ethanol^6^. Maintenance of cytosolic calcium levels by the action of Plasma Membrane Calcium ATPase (PMCA) is complemented by the joint participation of the Sarcoplasmic Endothelial Reticulum Calcium ATPase (SERCA2) for calcium uptake by the reticulum, and the Sodium Calcium Exchanger isoform 1 (NCX1) by calcium extrusion from cytoplasm to extracellular medium. It has been documented that in AP there is a lower expression of SERCA2 and NCX1 associated with calcium overload^13^. 

As intracellular calcium homeostasis is related to apoptosis, in the presence of calcium overload, drugs acting on calcium extrusion could be a strategy to minimize acinar pancreatic injury, targeting the sodium-calcium exchanger. In mice islet cell transplantation, the use of a sodium-calcium exchanger specific blocker, which blocks the reverse mode of the exchanger and allows calcium to migrate to the intracellular space, resulted in protection of the transplanted islets^15^.

The sodium-calcium exchanger is a carrier protein that contributes in intracellular calcium homeostasis in various cell types, having already been identified in the heart, kidney, aorta, brain, liver and pancreas^13^. It acts bi-directionally, with output of a calcium ion concomitantly with the entrance of three sodium ions or, when in the reverse mode, with the output of a sodium ion concurrently at the entrance of a calcium ion, through an electrogenic process^7^. In this process, there is NCX1 inactivation with changer drop in its trading activity until it reaches a steady state with the increase of intracellular sodium concentration. This process is called regulation or intracellular sodium-dependent inactivation, being mediated by an endogenous region called XIP (Exchange Inhibitor Peptide) formed by amino acids located at the N-terminal portion of the changer^19^. Studies on low molecular weight heparin action on rabbit aorta have identified the binding site of these fragments with the sodium-calcium exchanger in the XIP, which determines the output of intracellular calcium. In this situation, XIP inhibition by heparin fragments such as the trisulfate disaccharide (TD), favors the action of the sodium-calcium exchanger in cytoplasmic calcium removal in ion overload^19^.

In previous studies, on an experimental model of liver ischemia and reperfusion, we have demonstrated n TD hepatoprotective action with ALT and AST decrease and reduction of cellular necrosis^8^, as well as its “in vitro” action inducing extraction of intracellular calcium to the extracellular space in hepatocytes submitted to calcium overload situations^9^. The TD used in this research was the heparin fragment, so far without commercial formulation or approval for human use, but approved for experimental use, on this study^16^. 

On the other hand, experimental studies have demonstrated that melatonin, a hormone secreted by the pineal gland and the gastrointestinal tract^1,2 )^and possessing an antioxidant action, has a beneficial effect on AP^11^ ^,^ ^12^.

Therefore, we hypothesized that melatonin, by its antioxidant and cell protective effects^3^ and TD, by its action in accelerating the cytoplasmic calcium extraction through NCX1 could possibly modify the cellular lesion of AP, when used isolated or in combination, with a possible catalyzing effect. 

The objective of this study was to evaluate the expression of m-RNA of SERCA2 and NCX1 in an experimental taurocholate model of AP in rats pre-treated with melatonin and Trisulfate Disaccharide, isolated and combined.

## METHOD

The research was conducted in the Laboratory of Medical Investigation (LIM/37), Discipline of Transplant and Liver Surgery, Department of Gastroenterology, Faculty of Medicine, University of São Paulo, São Paulo, SP, Brazil.

### Experimental animals 

Twenty-five Wistar male rats, provided by the School of Medicine of the University of São Paulo, were used in this experiment, after approval by the Ethics Committee on animals the use (CEUA) of the Institution (Research Protocol nº 134/14). The rats were kept in individual cages in a temperature-controlled environment between 20-22^o^ C, fed with Nuvilab CR1 (Nuvital Nutrients LTDA) and hydrated with purified water ad libitum*.*

### Induction of acute pancreatitis 

Animals were anesthetized with 50 mg/kg of intraperitoneal ketamine hydrochloride 5% (Ketalar R, Cristália) and 10 mg/kg of 2% xylazine hydrochloride (Rompum R, Bayer) and heated by means of a 45W and 27V halogen lamp. Animal’s body temperature was kept between 35-37^º) )^C and monitored by a digital rectal thermometer (YSI Precision 4000A Thermometer, USA). AP was then induced by retrograde intraductal infusion of 0.5 ml of 3% sodium taurocholate into the pancreatic duct over one-minute period with a continuous flow infusion pump (KD Scientific, Holliston, MA). The proximal segment of the hepatic duct was clamped throughout the infusion period. Animals were kept in a temperature-controlled environment between 22-24º C for 2 h after the induction of AP before the collection of biological materials. 

They were divided into five groups: 1) Control Group (n=5): animals undergoing surgical manipulation of abdominal viscera, without induction of AP; 2) Group AP (n=5): animals submitted to AP without pre-treatment; 3) Group AP+Mel (n=5): animals receiving 50 mg/kg of melatonin, intraperitonially, 30 min before the induction of AP; 4) Group AP+TD (n=5): animals receiving 0.2 mg/kg of DT (0.4 ml injection into the penile vein), 10 min before the induction of AP; 5) Group AP+Mel+TD (n=5): animals submitted to AP, that received 0.2 mg/kg of DT (0.4 ml injection into the penile vein), 10 min before the induction of AP and 50 mg/kg of melatonin, intraperitonially, 30 min before the induction of AP.

### Material collection for analysis

Two hours after AP induction animals were anesthetized (40 mg/kg of ketamine 5%) for pancreatic tissue samples collection. Samples were stored in a freezer at -80° C until the time of RNA extraction procedure. 

After sacrifice, animal’s disposal was made in plastic bags and incinerated according to institution’s guidelines.

### Evaluation of NCX1 and SERCA2 gene expressions 

Total RNA was extracted from pancreatic tissue frozen samples by using TRIzol reagent (Invitrogen Life Technologies)™, according to manufacturer’s instructions. Extracted total RNA samples were quantified in Nanodrop spectrophotometer-1000 and the evaluation of the integrity of total RNA extracted in 1% agarose gel. After quantification and electrophoresis, only total RNA samples with reason 260/280 nm ≥1.8 and visualization of bands characteristics 18S and 28S were considered viable. The program Prime 3 (http://primer3.ut.ee) was used to obtain the oligonucleotides design for the qRT-PCR reactions procedures. The oligonucleotides sequences were thus determined:

NCX1 (forward): CACCTGTGGAGAGCTGGAAT
NCX1 (reverse): AGACGGGGTTCTCCAATCTC
SERCA2 (forward): TCGAAGAAGGTGAAGAAACGA
SERCA2 (reverse): CTTGCCCATTTCAGGTTCAT
Beta ACTIN (forward): TGTCACCAACTGGGACGATA
Beta ACTIN (reverse): GGGGTGTTGAAGGTCTCAAA

The analysis of m-RNA levels expressions of NCX1 and SERCA2 genes was held in thermal cycler Rotor-Gene RG-3000 (Corbett Research, Sydney, Australia). The commercial kit SuperScript™ III Platinum^®)^ SYBR Green one-step qRT-PCR (Invitrogen, Life Technologies) was used, which corresponds to the reaction performed in a single step, where the synthesis of cDNA was performed from total RNA sample. For that, it was used for a final volume of 15 µl: 0.3µl of enzyme, 7.5µl of buffer, 0.3µl of oligonucleotide forward, 0.3µl of oligonucleotide reverse, 1.6 µl of DNase/RNase free sterile ultrapure water and 5 µl of total RNA (20ng). All samples were done in duplicate. For the internal control of qRT-PCR reactions the reference gene (endogenous) beta-ACTIN was used to normalize the results obtained for the target genes. 

For determination of each target gene expression level, the 2-delta delta Ct method for quantification^14^ was used, where Ct (threshold cycle) is the real-time PCR cycle, when amplification reaches the logarithmic phase, where delta Ct is the difference between target gene expression and endogenous control of a given sample and delta Ct corresponds to the difference between the delta Ct sample and the delta Ct control.

### Statistical Analysis

Quantitative variables with averages and standard deviation were evaluated by variance analysis test. Graph Pad Prism version 6.0 program was used for the statistical analysis and a significance level of p<0.05 was accepted.

## RESULTS

The expressions of SERCA2 and NCX1 decreased significantly in the AP Group when compared to the control group (Table 1).

**TABLE 1:** Expressions of SERCA2 and NCX1 in the AP Group compared to the control group.

Control	SERCA2	NCX1	AP	SERCA2	NCX1
1	1.77	1.55	1	0.12	0.33
2	1.57	1.32	2	0.34	1.2
3	1.5	1.44	3	0.23	0.18
4	1.46	1.04	4	0.14	0.34
5	1.23	1.84	5	0.12	0.15
MEAN	1.506	1.438	MEAN	0.19	0.44
SD	0.195	0.294	SD	0.095	0.433

AP=acute pancreatitis; SERCA2=p<0.008; NCX1=p<0.01.

There were no significant changes in SERCA2 and NCX1 expressions in rats of the Group AP + TD when compared with the AP Group (Table 2).

**TABLE 2:** Expressions of SERCA2 and NCX1 in AP+TD Group in relation to the AP Group

AP	SERCA 2	NCX1	AP+DT	SERCA 2	NCX1
1	0.12	0.33	1	0.17	0.2
2	0.34	1.2	2	0.13	0.2
3	0.23	0.18	3	0.15	0.3
4	0.14	0.34	4	0.12	0.13
5	0.12	0.15	5	0.12	0.43
MEAN	0.19	0.44	MEAN	0.138	0.252
SD	0.095	0.433	SD	0.022	0.116

AP=acute pancreatitis; TD=acute pancreatitis + trisulfate disaccharide; SERCA2=p<0.59; NCX=p<0.60

SERCA2 expression was increased in Group AP + Mel when compared to the AP Group. There was no significant difference in NCX1 expression between the two groups (Table 3).

**TABLE 3:** Expressions of SERCA2 and NCX1 in Group AP + Mel in relation to the AP Group

AP	SERCA 2	NCX1	AP+MEL	SERCA 2	NCX1
1	0.12	0.33	1	0.45	0.2
2	0.34	1.2	2	0.43	0.19
3	0.23	0.18	3	0.34	0.15
4	0.14	0.34	4	0.63	0.31
5	0.12	0.15	5	0.26	0.27
MEAN	0.19	0.44	MEAN	0.422	0.224
SD	0.095	0.433	SD	0.139	0.065

AP=acute pancreatitis; AP+ MEL=AP+melatonin; SERCA2=p<0.02; CX1=p< 0.40

No significant alteration of SERCA2 expression and a significant reduction of NCX1 expression were observed in Group AP+Mel+DT, when compared to Group AP (Table 4). 

**TABLE 4:** Expressions of SERCA2 and NCX1 in Group AP+MEL+DT in relation to the AP Group

AP	SERCA 2	NCX1	AP+MEL+TD	SERCA 2	NCX1
1	0.12	0.33	1	0.17	0.07
2	0.34	1.2	2	0.15	0.1
3	0.23	0.18	3	0.32	0.13
4	0.14	0.34	4	0.21	0.29
5	0.12	0.15	5	0.31	0.2
MÉDIA	0.19	0.44	MÉDIA	0.232	0.158
DP	0.095	0.433	DP	0.079	0.088

AP=acute pancreatitis; AP+MEL+DT=AP+melatonin+trisulfate disaccharide; SERCA2=p<0.34; NCX1=p< 0.07

In Group AP+Mel+DT there was a significant decrease of SERCA2 expression in relation to NCX1 expression, when compared to the Group AP+Mel (Table 5).

**TABLE 5:** Expressions of SERCA2 and NCX1 in Group AP+Mel+TD in relation to Group AP+Mel

AP+MEL	SERCA 2	NCX1	AP+MEL+TD	SERCA 2	NCX1
1	0,45	0,2	1	0,17	0,07
2	0,43	0,19	2	0,15	0,1
3	0,34	0,15	3	0,32	0,13
4	0,63	0,31	4	0,21	0,29
5	0,26	0,27	5	0,31	0,2
MÉDIA	0,422	0,224	MÉDIA	0,232	0,158
DP	0,139	0,065	DP	0,079	0,088

AP+MEL=acute pancreatitis+melatonin; AP+MEL+DT=acute pancreatitis+melatonin+trisulfate disaccharide; SERCA2=p< 0.03; NCX1=ns

In animals of group AP+Mel there was a significant increase in SERCA2 expression when compared to the Group AP+TD. There was no difference in the change in NCX1 expression between the two groups (Table 6).

**TABLE 6:** Expressions of SERCA2 and NCX1 in Group AP+Mel in relation to Group AP+DT

AP+TD	SERCA 2	NCX1	AP+MEL	SERCA 2	NCX1
1	0,17	0,2	1	0,45	0,2
2	0,13	0,2	2	0,43	0,19
3	0,15	0,3	3	0,34	0,15
4	0,12	0,13	4	0,63	0,31
5	0,12	0,43	5	0,26	0,27
MÉDIA	0,138	0,252	MÉDIA	0,422	0,224
DP	0,022	0,116	DP	0,139	0,065

AP+DT=Acute pancreatitis+trisulfate disaccharide; AP+Mel=acute pancreatits+melatonin; SERCA2=p< 0.01; NCX1=ns

## DISCUSSION

Since calcium overload is possibly involved in tissue injury in AP, the use of drugs that act on this element may be beneficial in reducing the inflammatory process of this disease. On the other hand, drugs with different action mechanisms may eventually act synergistically with this purpose. Therefore, trisulfate disaccharide (TD), which is active in removal of intracellular calcium^9^ and melatonin, an antioxidant agent^13^, theoretically could act synergistically in this situation. The purpose of the present study was to verify this possible synergy, aiming at a better understanding of the phenomena involved in cellular injury in a model of acute experimental pancreatitis. 

Considering the joint participation of SERCA2 and NCX1 in calcium kinetics and the reduction of their expressions in AP associated with calcium overload^13^, in this study m-RNA expressions of SERCA2 and NCX1 were analyzed in animals submitted to AP by taurocholate with and without pre-treatment with melatonin and DT, isolated and in association.

Confirming data from previous reports^13^, the present study showed that experimental AP leads to a marked reduction of SERCA2 and NCX1 expressions. The reversal of this expression decrease, as well as the increased expression of m-RNA of SERCA2 in the melatonin pretreatment group (Group AP+Mel), even without increase in NCX1 expression, suggests a possible pharmacogenic beneficial effect of melatonin. However, increased expressions of SERCA2 and NCX1 were not observed in animals pretreated with TD either isolated or associated with melatonin. In fact, when administered with melatonin, TD determined a decreased SERCA2 expression, when compared to isolated melatonin pre-treatment. 

This result is probably due to the increased intracellular calcium output caused by the TD action on NCX1, accelerating its function in calcium overload situations. Consequently, the absence of an increased SERCA2 expression could be due to the action balance between NCX1 and SERCA2, with augmented function of existing NCX1, reduces the need for SERCA2 action for calcium transport to the endoplasmic reticulum.

This suggests the existence of two different mechanisms involved in the process: the increase of SERCA2 gene expression caused by melatonin, without acting in NCX1, and the TD action on the function of remaining cellular structures after the AP cell injury, increasing the NCX1 activity, but without a pharmacogenic action on the involved structures. Maintaining the viability of cell structures and their functions, by its antioxidant effects, melatonin could facilitate joint action of other substances, with different mechanisms of action, in the preservation of pancreatic tissue injured in AP. 

Considering the important findings of this research, other studies are required for a better understanding of the mechanisms involved in the actions of different drugs in this situation. 

## CONCLUSION

The effect of melatonin is restricted to increased expression of SERCA2. TD has no action on gene expression; however, its action on accelerating the sodium calcium exchanger in calcium withdrawal may explain the lower expression of SERCA2 when associated with melatonin, by the joint action of drugs with different and possibly complementary mechanisms.
